# Strategy for ipsilateral multiple concomitant upper extremity fractures in an elderly polytraumatized patient with severe osteoporosis: A case report

**DOI:** 10.1097/MD.0000000000041958

**Published:** 2025-08-01

**Authors:** Bum-Jin Shim, Dae Hee Lee, Hong Bae Jeon, Yong Chan Kim, Kyung Wook Kim

**Affiliations:** a Department of Orthopedic Surgery, Yeungnam University Hospital, Yeungnam University College of Medicine, Daegu, South Korea; b Department of Orthopaedic Surgery, Dankook University Hospital, Dankook University College of Medicine, Cheonan, South Korea; c Department of Plastic and Reconstructive Surgery, Dankook University Hospital, Dankook University College of Medicine, Cheonan, South Korea.

**Keywords:** concomitant fractures, elderly, osteoporosis, polytrauma, traffic accident

## Abstract

**Rationale:**

Elderly patients with severe osteoporosis present significant challenges in managing multiple concomitant upper extremity fractures, especially when high-energy trauma is involved. The rarity of such cases necessitates a structured approach to achieve optimal outcomes, balancing surgical intervention and early rehabilitation.

**Patient concerns:**

A 79-year-old woman presented to the emergency department following a severe car accident. She had multiple concomitant fractures, including those of the proximal humerus, proximal ulna, and open fractures of the radius in the ipsilateral upper extremity, as well as multiple organ injuries involving the brain, lung, and liver.

**Diagnoses:**

The patient was diagnosed with an open fracture of the right proximal radius, a comminuted intra-articular fracture of the proximal ulna, and comminuted intra-articular fractures of the right proximal humerus involving the glenoid and coracoid. Additionally, non-displaced fractures of the left distal clavicle and left sacral body, and minimally displaced fractures of both the superior and inferior rami of the pelvis were confirmed.

**Interventions:**

The patient underwent hemiarthroplasty for the shoulder fracture due to severe comminution, open reduction and internal fixation for the proximal ulna, and intramedullary nailing for the radius. Post-operative management included the sequential administration of teriparatide and denosumab to support bone healing and facilitate early rehabilitation.

**Outcomes:**

The patient had a relatively short hospital stay of 5 days and showed favorable outcomes, with early callus formation and bone union confirmed at 6 months. At the 1-year follow-up, the patient demonstrated satisfactory joint stability and improved bone mineral density.

**Lessons:**

This case highlights the potential of combined surgical approaches and bone-strengthening therapies in achieving effective outcomes in elderly polytraumatized patients. Sequential administration of teriparatide and denosumab may be beneficial for promoting bone healing in severe osteoporotic fractures.

**Level of evidence: Level V, case report.**

## 1. Introduction

Recently, the number of elderly patients with upper extremity fractures has been increasing with the aging of the population.^[1]^ Due to the osteoporotic nature of their bones, even those with ground-level falls can experience upper extremity fractures, including proximal humerus fractures. Geriatric osteoporotic fractures with high-energy injuries are associated with high morbidity and mortality rates. The treatment presents significant challenges, particularly in multiple concomitant fractures of the upper extremities.^[2,3]^

Early management of fractures, either by open reduction and internal fixation (OR/IF) or arthroplasty, has become the gold standard for promoting early rehabilitation, aiming to reduce morbidity and enable return to ordinary life. However, elderly patients with multiple fractures experience treatment difficulties and a prolonged length of hospital stay. Therefore, they pose socioeconomic burdens and challenges in surgical management and recovery.

In the current report, we present a case of high-energy ipsilateral injury with concomitant shoulder and elbow fractures in a polytraumatized elderly patient. We treated patients with a combination of arthroplasty, OR/IF, and closed reduction (CR)/IF, which have shown reliable outcomes despite severe multiple injuries. Through this case report, we aimed to increase awareness of geriatric concomitant upper extremity fractures, which are becoming more common, and to provide treatment options to reduce the length of hospital stay and morbidity.

## 2. Case report

This study’s design was approved by the institutional review board of the Dankook University College of Medicine/Dankook University Hospital (No. 2023-08-012). Moreover, the written informed consent was obtained from patient. A 79-year-old woman was transferred to a tertiary referral hospital with a level I trauma center following of a serious car accident. She had a past medical history of medically uncontrolled hypertension. Her hemodynamics deteriorated, with a pulse rate of 110 beats per minute and a systolic blood pressure of 80 mm Hg on arrival. On initial examination, a brain computed tomography (CT) scan showed traumatic subdural and falx hemorrhages, while abdominal CT showed grade II liver injury (Seg. V and VII). However, none of the injuries required surgical treatment. Otherwise, the chest CT scan showed a massive pulmonary contusion with traumatic hemothorax in the left lung and multiple rib fractures on both sides (Rt., 4–11th; Lt., 4–8th); therefore, thoracostomy was performed for the left hemothorax in the emergency room.

**Figure 1.**
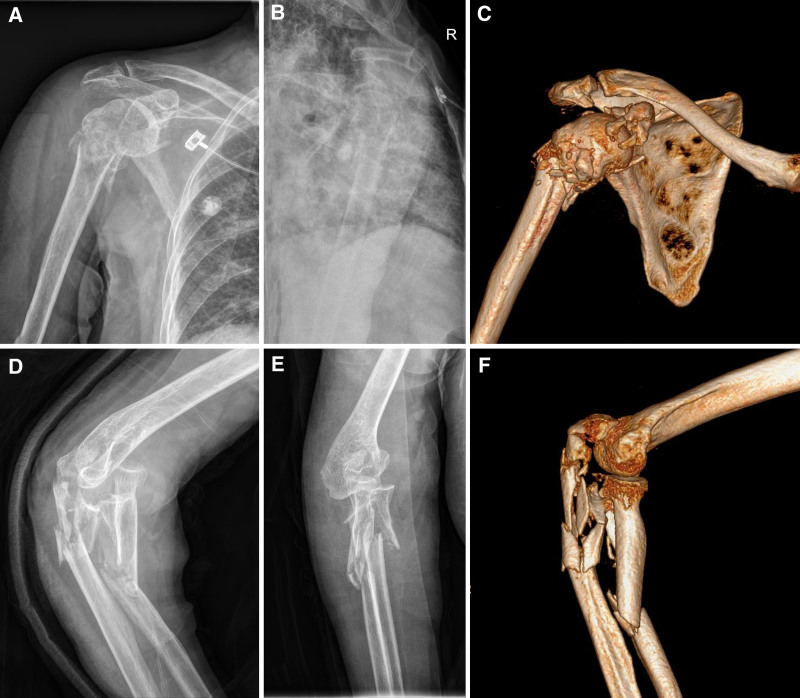
Patient’s initial pre-operative plain radiographs and computed tomography (CT). (A–C) The patient sustained comminuted intra-articular fractures of the right proximal humerus (AO/OTA fracture type C3), and fractures of the glenoid and coracoid. (D–F) An open fracture of the right proximal radius (2R2B) and comminuted intra-articular fracture of the proximal ulna (2U1C) were confirmed. AO/OTA = Arbeitsgemeinschaft für Osteosynthesefragen Foundation/Orthopaedic Trauma Association.

After initial supportive treatment, plain radiographs and CT scans of the injured limbs were evaluated. The patient sustained an open fracture of the right proximal radius (Arbeitsgemeinschaft für Osteosynthesefragen Foundation/Orthopaedic Trauma Association fracture type 2R2B), comminuted intra-articular fracture of the proximal ulna (2U1C), comminuted intra-articular fractures of the right proximal humerus (C3), and fractures of the glenoid and coracoid (Fig. 1). Additionally, a non-displaced fracture of the left distal clavicle and left sacral body, and minimally displaced fractures of both the superior and inferior rami of the pelvis were confirmed. After confirmation of the pre-operative assessment by trauma center specialists and an anesthesiologist, the patient underwent surgery within 36 hours of injury on the day of admission.

**Figure 2.**
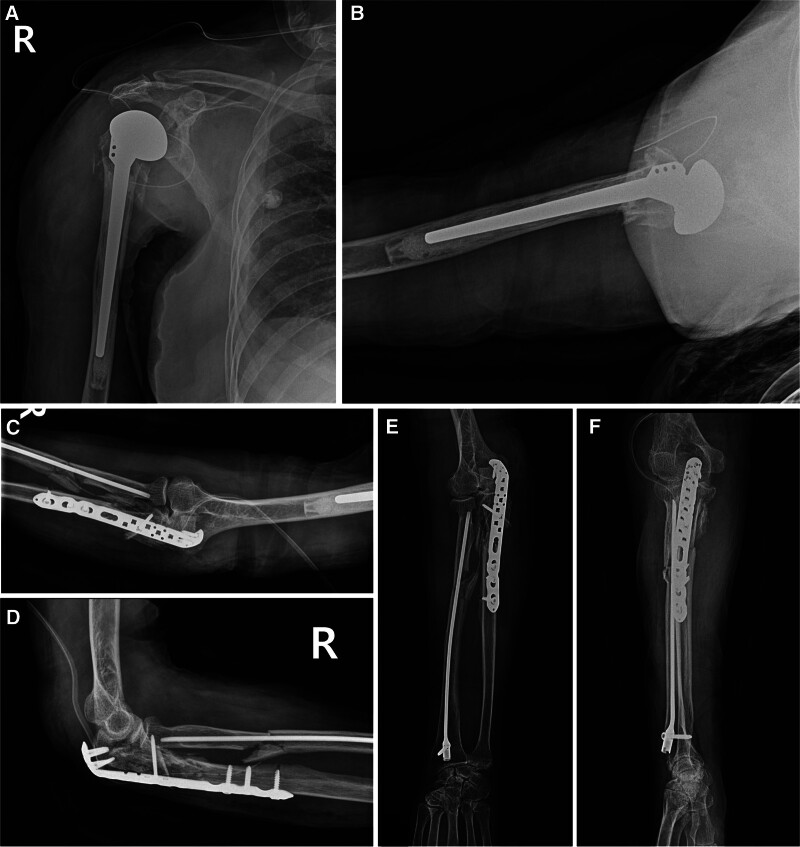
Patient’s immediate post-operative plain radiographs. (A, B) Bipolar hemiarthroplasty for the shoulder fracture dislocation was performed. (C–F) Open reduction and internal fixation were performed for the proximal ulna fracture. However, the skin condition of the anterior side of the forearm was poor due to the open wound at the fracture site, and to shorten the surgery time, intramedullary nail fixation was performed for the proximal radius fracture.

First, for the shoulder joint fractures, reverse total shoulder arthroplasty was planned, considering the severity of the fracture, patient age, and the risk of avascular necrosis. However, due to severe comminution of the glenoid, insertion of the baseplate was thought to be difficult. Hence, hemiarthroplasty was performed using a Comprehensive ® Fracture Stem (Biomet, Warsaw). Subsequently, owing to the severe comminution of the ulna, the radius was operated first, to match the length and alignment of the forearm bone. Furthermore, the skin condition of the anterior side of the forearm was poor due to the open wound at the fracture site and multiple abrasions. To shorten the surgery time, intramedullary nail fixation (Acumed, Hillsbrough) was performed. Since the comminution of the fracture site of the proximal ulna was severe, bridging plating was performed using a Variable-Angle LCP® Olecranon Plate (DePuy Synthes, Oberdorf, Switzerland) after conforming articulation of the articular surface of the olecranon (Fig. 2).

An extended long-arm splint was applied for the first 2 post-operative weeks. Two weeks after surgery, an extended long-arm removable splint was applied for the following 4 weeks with the recommendation of night use, and physical therapy was initiated to ensure protected active motions. This exercise progressed to active assistive motion after one month. Postoperative bone mineral density was assessed using dual-energy X-ray absorptiometry. The T-score at the L3–L4 lumbar spine and femoral neck were −3.4 and −1.2, respectively. Therefore, in this patient with severe osteoporosis, teriparatide (Forsteo®) was used for the first 3 months and then switched to denosumab (Prolia®) to promote bone union.

**Figure 3.**
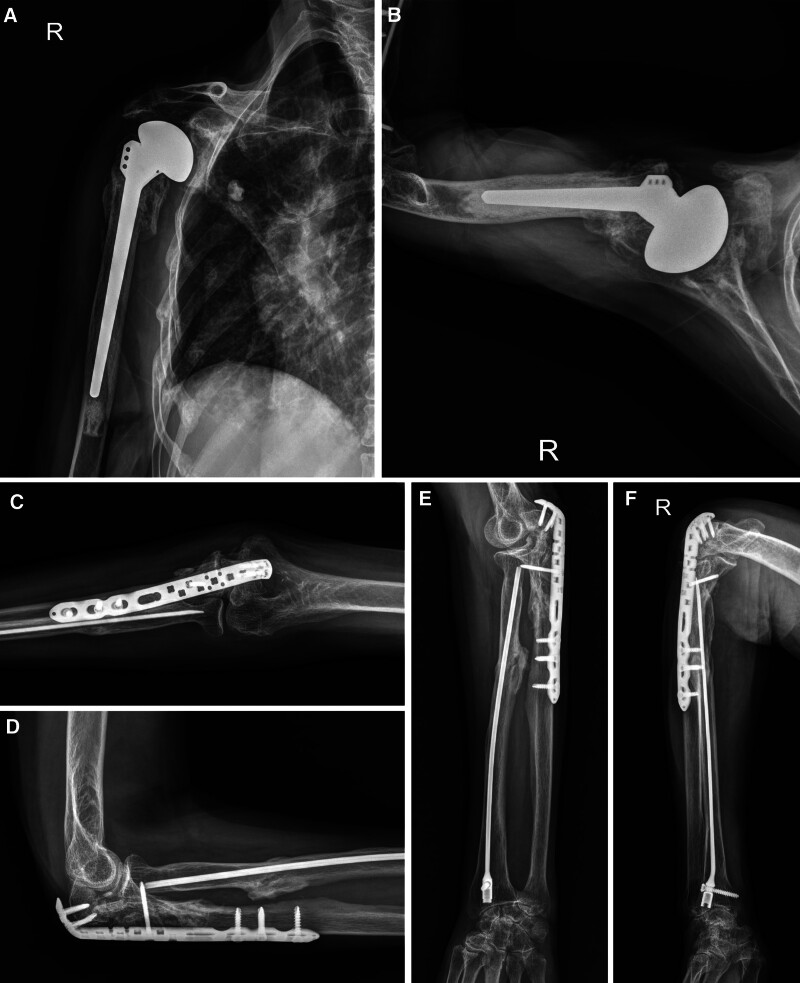
Patient’s post-operative plain radiographs at six months. (A, B) At 6 months, bipolar hemiarthroplasty was well maintained. (C–F) Open or closed reduction and internal fixation for the proximal ulna and radius fractures were also well maintained. Solid bone union was achieved and there were no complications at six months.

**Figure 4.**
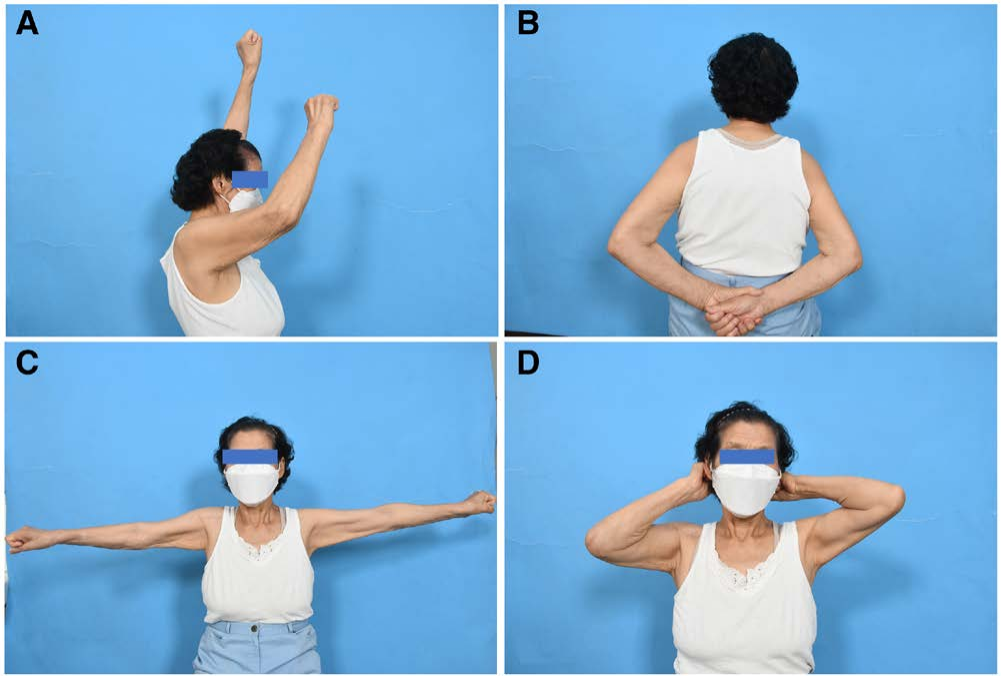
Post-operative clinical photographs of patient. (A–D) Satisfactory range of motion of the shoulder and elbow joints was achieved at the final follow-up.

Despite the severity of the injuries, the patient had a relatively short length of hospital stay of five days, and the rehabilitation course was favorable without any complications. From the ulna at the 6th week to the radius at the 8th week, callus formation was observed at the fracture sites. Subsequently, bone union was confirmed in all bones at six months (Fig. 3). Furthermore, the T-scores improved to −2.3 and −0.9, respectively, at the 1-year follow-up. The shoulder and elbow joints were stable without any dynamic instability. At the 1-year post-operative follow-up, there was no limitation of range of motion in the elbow joint, but arthritic changes and limited motion occurred in the shoulder joint (Fig. 4). However, the patient did not complain of any functional limitation in daily activities or instability of the shoulder joint. Additionally, the function of the fractured limb was evaluated using the disabilities of the arm, shoulder, and hand score, which was 10.3 at the 1-year post-operative follow-up.

## 3. Discussion

Surgery for multiple ipsilateral concomitant upper extremity fractures, especially in elderly polytraumatized patients with osteoporosis, is difficult and challenging. In the current case, we treated the proximal humeral fractures with hemiarthroplasty, OR, and CR/IF for proximal ulnar and radius fractures. Consequently, our results demonstrate reliable clinical and radiological outcomes.

Concomitant proximal humerus, proximal ulna, and radius open fractures of the ipsilateral upper extremities are relatively rare in elderly patients.^[3]^ Although this patient had a high-energy injury, it was probably related to patient’s preexisting osteoporosis. In this case, the shoulder joint was unstable due to the fracture and dislocation, and the elbow joint was unstable due to the proximal radius and ulnar comminuted open fractures and dislocation. Therefore, conservative treatment is challenging; surgical treatment is mandatory, and early rehabilitation is required.^[4]^

There is no consensus regarding the order of surgery for multiple fractures of the ipsilateral upper extremities.^[5,6]^ In the current case, the shoulder joint was initially treated because of its instability. We considered that once the shoulder joint was stabilized, the OR/IF of the proximal radius and ulna would be substantially more appropriate.

Most proximal humeral fractures in elderly patients with osteoporosis are low-energy injuries. Therefore, conservative treatment is commonly considered according to the fracture pattern. Moreover, there is no difference in clinical outcomes between conservative treatment and surgery for proximal humeral fractures with osteoporosis.^[7]^ However, this patient had a high-energy injury with fracture-dislocation and was unstable. Although OR/IF can be attempted in elderly patients with fracture-dislocation, there is a risk of avascular necrosis and implant failure. Additionally, the surgery was delayed for 3 days because the patient’s general condition was unstable due to multiple organ injuries, including brain, liver, and lung. In addition, if the proximal humeral fracture had been operated on by OR/IF, rehabilitation would have been delayed compared with arthroplasty. To facilitate early rehabilitation and eliminate the possibility of a second surgery (conversion to arthroplasty), the decision was made to opt for primary arthroplasty.^[8]^

Regarding the treatment options for radial fractures of the diaphysis, internal fixation with a plate can provide exact anatomic reduction with restoration of radial bowing compared with intramedullary nailing (IMN).^[9]^ However, several studies have not shown significant differences in functional outcomes between OR/IF with plates and IMN. In the current case, in addition to a Gustilo-Anderson grade 2 open comminuted intra-articular fracture of the right proximal radius, the patient had multiple abrasions and contusions on the anterior aspect of the forearm. Hence, the wound was unsuitable for an open incision, and considering the patient’s overall condition, we performed CR/IF with IMN for the proximal radius fracture and OR/IF with a plate for the proximal ulnar fracture. Bone union was achieved without complications.

Several studies revealed that the administration of teriparatide which is the recombinant human parathyroid hormone can promote the healing of fracture.^[10–13]^ Therefore, considering that the patient is a post-menopausal woman with osteoporosis, we decided to administrate teriparatide (Forsteo®). However, to date there is no consensus on the duration of drug use. Therefore, we used it for up to three months until sufficient callus formation was confirmed, and then switched to denosumab (Prolia®). As a result, when the first one year after administration, we could find that the bone union of fracture sites was confirmed, and bone mineral density was also improved. Hence, the administration of teriparatide may be a reliable option for promoting bone healing in post-menopausal women with severe osteoporosis.

The length of hospital stay is a crucial metric for assessing trauma care.^[14]^ This patient was discharged five days after surgery. Nevertheless, no complications, including infection, occurred during outpatient clinic follow-up. In addition, rehabilitation proceeded sequentially. This expedited discharge resulted in significant cost savings for the patient. Therefore, our case showed excellent results in overall aspects, including surgery time, clinical results, radiological results, and cost. We believe that this could be achieved through appropriate surgical management.

Although the current case has rarely been reported and it is difficult to generalize, we believe it is a meaningful report in that it presents a viable option for the challenging treatment of ipsilateral multiple concomitant upper extremity fractures in an elderly patient with severe osteoporosis.

## 4. Conclusion

Multiple concomitant upper extremity fractures in elderly polytraumatized patients with osteoporosis are relatively rare and challenging to treat, and more may result in increased morbidity and length of hospital stay. Therefore, in addition to adequate operative management of the fractures, sequential administration of teriparatide and denosumab may be a viable option for multiple concomitant upper extremity fractures in elderly polytraumatized patients with osteoporosis.

## Author contributions

**Conceptualization:** Bum-Jin Shim, Kyung Wook Kim.

**Data curation:** Dae Hee Lee.

**Investigation:** Hong Bae Jeon, Yong Chan Kim.

**Methodology:** Hong Bae Jeon, Yong Chan Kim.

**Supervision:** Bum-Jin Shim, Kyung Wook Kim.

**Writing – original draft:** Bum-Jin Shim, Dae Hee Lee.
